# Ropivacaine-based Regional Anesthesia Exerts Muscle-protective Effects Despite Elevated Compartment Pressure in a Porcine Model of Acute Compartment Syndrome

**DOI:** 10.1097/ALN.0000000000005950

**Published:** 2026-01-23

**Authors:** Gerhard Fritsch, Philipp Thaler, James Ferguson, Helena Thumfart, Johannes Grillari, Marcin Osuchowski, Gebhard Woisetschlaeger, Sandra Hoegler, Aniko Gutasi, Johannes Zipperle

**Affiliations:** 1 Department of Anesthesiology and Intensive Care Medicine, AUVA Trauma Center Salzburg, Academic Teaching Hospital of the Paracelsus Medical University, Salzburg, Austria; Ludwig Boltzmann Institute for Traumatology, the Research Center in cooperation with AUVA, Vienna, Austria; and Paracelsus Medical University, Salzburg, Austria.; 2 Ludwig Boltzmann Institute for Traumatology, the Research Center in cooperation with AUVA, Vienna, Austria.; 3 Ludwig Boltzmann Institute for Traumatology, the Research Center in cooperation with AUVA, Vienna, Austria.; 4 Ludwig Boltzmann Institute for Traumatology, the Research Center in cooperation with AUVA, Vienna, Austria.; 5 Ludwig Boltzmann Institute for Traumatology, the Research Center in cooperation with AUVA, Vienna, Austria; and Institute for Molecular Biotechnology, BOKU University, Vienna, Austria.; 6 Ludwig Boltzmann Institute for Traumatology, the Research Center in cooperation with AUVA, Vienna, Austria.; 7 Ludwig Boltzmann Institute for Traumatology, the Research Center in cooperation with AUVA, Vienna, Austria.; 8 University of Veterinary Medicine Vienna, Center for Pathobiology, Laboratory Animal Pathology, Vienna, Austria.; 9 Ludwig Boltzmann Institute for Traumatology, the Research Center in cooperation with AUVA, Vienna, Austria.; 10 Ludwig Boltzmann Institute for Traumatology, the Research Center in cooperation with AUVA, Vienna, Austria.

## Abstract

**Background:**

Acute compartment syndrome (ACS) is a devastating sequela of orthopedic trauma characterized by elevated compartment pressure that precipitates tissue ischemia. The role of regional anesthesia in modulating these pathophysiological events remains controversial. This study investigated the impact of regional anesthesia *via* ropivacaine 0.2% (RPVC) on compartment pressure, systemic hemodynamics, biochemical markers, and tissue integrity in a porcine model of ACS.

**Methods:**

Twenty male pigs underwent ACS induction in the tibialis anterior compartment by targeted inflation of an angioplasty catheter and were randomized to receive either RPVC or placebo *via* a regional catheter. Continuous compartment pressure and mean arterial pressure were recorded. Plasma samples and interstitial fluid were collected at predefined time points (T1, T3, T6, T8, and T10) to quantify creatine kinase, lactate, lactate dehydrogenase, and glucose. Histopathologic evaluation was performed at the end of the experiment (T10).

**Results:**

At T10, compartment pressure was significantly elevated in the RPVC group (57.0 mmHg [49.0 to 59.0]) compared to placebo (41.5 mmHg [38.2 to 47.2]; *P* = 0.022), whereas mean arterial pressure remained comparable (RPVC: 63.0 mmHg [58.0 to 65.0] *vs*. placebo: 59.0 mmHg [56.2 to 61.8]; *P* = 0.13). Plasma biomarkers did not differ significantly (*e.g.*, plasma creatinine kinase: RPVC 1940 U/l [868 to 3,334] *vs*. placebo 1,171 U/l [1,020 to 1,467]; *P* = 0.24). In contrast, interstitial analysis demonstrated a marked reduction in tissue injury markers in the RPVC group, with interstitial fluid lactate dehydrogenase significantly lower [24,475 U/l (19,500 to 28,213) *vs*. 113,800 U/l (91,000 to 116,600); *P* = 0.017] and interstitial fluid glucose substantially decreased (19.5 mg/dl [11.5 to 27.5] *vs*. 51.0 mg/dl [43.0 to 103.0]; *P* = 0.0043). RPVC-treated tissue exhibited attenuated degeneration and reduced necrosis in a blinded histopathologic scoring.

**Conclusions:**

Despite a higher compartment pressure, RPVC-based regional anesthesia yielded an improved regional metabolic profile and mitigated tissue injury in ACS. These findings suggest a protective effect in the observed timeframe that merits further clinical exploration.

## Abstract 2

This randomized controlled study in a porcine model of acute compartment syndrome explored regional anesthesia’s effects (ropivacaine *vs.* saline sciatic block) on blood flow, compartment pressures, tissue perfusion, metabolites, and histologic damage. Despite ropivacaine block being associated with higher intracompartmental pressures, it also was associated with superior overall perfusion, with lower interstitial fluid and histologic evidence of ischemia and necrosis. These findings support the previous clinical evidence suggesting that not only is regional anesthesia not harmful in acute compartment syndrome, but it may actually be beneficial.

## Editor’s Perspective

**What We Already Know about This Topic**

- Acute compartment syndrome (ACS) is a serious complication of trauma caused by elevated muscle pressure, leading to tissue damage and possible limb loss
- While regional anesthesia use is debated in patients at risk of ACS, due to concerns about masking pain, it may improve blood flow and influence ACS progression

**What This Article Tells Us That Is New**

- This randomized controlled study in a porcine model of acute compartment syndrome explored regional anesthesia’s effects (ropivacaine *vs.* saline sciatic block) on blood flow, compartment pressures, tissue perfusion, metabolites, and histologic damage
- Despite ropivacaine block being associated with higher intracompartmental pressures, it also was associated with superior overall perfusion, with lower interstitial fluid and histologic evidence of ischemia and necrosis
- These findings support the previous clinical evidence suggesting that not only is regional anesthesia not harmful in ACS, but it may actually be beneficial

Acute compartment syndrome (ACS) is among the most serious complications after orthopedic trauma. It is defined by an elevation of pressure within a muscular compartment leading to cellular swelling, edema, and, if untreated, cell death and necrosis. Although the incidence of ACS is low, it can result in functional impairment and even loss of the affected limb. Forearm fractures and diaphyseal tibial fractures are the most common injuries associated with ACS.^1–7^ The primary pathophysiological mechanisms of ACS are arteriovenous perfusion mismatch and ischemia–reperfusion injury, both of which contribute to elevated compartment pressure.^3^ Young athletic males sustaining high-speed injuries are particularly at risk.^5,7^ Although nontraumatic causes such as metabolic changes, thermal injuries, and neurologic emergencies (*e.g.*, intoxication and coma) can also trigger ACS, these etiologies are relatively rare.

Apart from clinical examination and the subjective perception of pain, invasive measurements of compartment pressure are recommended to confirm and monitor ACS.^1–3,8^ In healthy individuals, compartment pressure is typically less than 10 mmHg, but a differential pressure (mean arterial pressure minus compartment pressure) less than 30 mmHg is considered the most reliable indicator of ACS.^1,8–11^

Regional anesthesia has become a widely used method of pain management in orthopedic patients. By providing effective pain relief, regional anesthesia also reduces the need for opioid administration. However, the use of regional anesthesia in patients at risk for ACS remains controversial. Some reports suggest that regional anesthesia can delay the diagnosis of ACS, while others find no significant interference.^12–18^ The regional anesthesia–related debate often reflects the perspectives of different medical specialties. Orthopedic surgeons are more cautious about recommending regional anesthesia, while anesthesiologists are inclined to support its use, believing that ischemic breakthrough pain would remain detectable.^12,19^

While most studies have focused on whether regional anesthesia delays ACS diagnosis, the potential influence of regional anesthesia on the progression of ACS has been largely unexplored. There is evidence that regional anesthesia alters hemodynamics in the blocked regions demonstrated by studies on axillary, supraclavicular, and sciatic nerve blocks. Those studies have shown that regional anesthesia is associated with elevated perfusion indices (*i.e.*, the difference between peak systolic and diastolic blood flow as measured by pulse wave analysis) in the affected limbs. This suggests that regional anesthesia exerts favorable effects on perfusion, which in turn could influence the development and clinical course of ACS.^20–22^

Given the potential effects of regional anesthesia on perfusion, we conducted a prospective, randomized, preclinical study in a porcine model to assess the influence of regional anesthesia on the development and progression of ACS. To enable this investigation, we adapted and refined an established porcine ACS model, incorporating continuous local anesthetic infusion and interstitial microperfusion sampling for detailed tissue-level analysis. Although such animal models cannot fully replicate human physiology or clinical complexity, they provide essential mechanistic insights that form the basis for future translational research.

## Materials and Methods

### Animals

A total of 20 male house race pigs (*Sus scrofa domesticus*), from a local breeder in Lower Austria, Austria, weighing 35,70 ± 2,81 kg were included in a standardized setup to induce ACS in the lower extremity and to test the effect of regional anesthesia in comparison with a placebo group. Only male pigs were used, as ACS predominantly affects young males, reflecting the clinical population most at risk.^23^ The animals were housed according to the standard operating procedures of the host institution. One to three days before the experiments, the animals were transferred to the surgical unit’s stable. During this period, they were provided with a diet in accordance with the guidelines of the breeding institution. Environmental enrichment, including straw, grass, and rubber toys, was provided to promote animal well-being. Food and water were offered *ad libitum*, and continuous veterinary surveillance was ensured. At the end of the experiment, the animals were humanely euthanized under deep anesthesia through the administration of a lethal dose of pentobarbital.

The study adhered to Austrian and European animal welfare guidelines, with strict compliance to European Union directive No. 2010/63/EU concerning the use of animals for scientific purposes. All animal experiments were performed in the Ludwig Boltzmann Institute for Traumatology, Vienna, Austria, under the approval of the local legislative ethical committee (permit No. MA58-2348738-2022-12) and conducted observing National Institutes of Health guidelines.

### Randomization and Treatment Groups

**Fig. 1.**
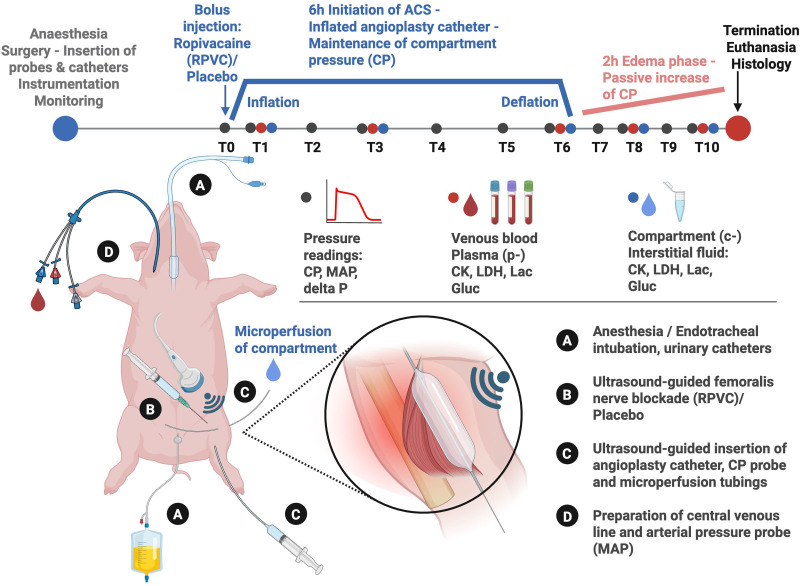
Timeline and experimental setup of the porcine model of ACS, modified after Kalns *et al.*^24^ Sampling times and endpoints (T1 to T10), preparation procedures and interventions (*A* to *D*) are displayed schematically and are briefly described at the bottom right. ACS, acute compartment syndrome; c-, compartment; CK, creatine kinase; CP, compartment pressure; Gluc, glucose; Lac, lactate; LDH, lactate dehydrogenase; MAP, mean arterial pressure; p-, plasma; RPVC, ropivacain.

Randomization was performed using the web-based software Research Randomizer (version 4.0; https://www.randomizer.org) before study initiation. The animals were randomly allocated to one of two groups using a simple randomization protocol, and two animals underwent surgery in parallel. All participating staff was blinded to the group assignments until the finalization of analyses. An individual not otherwise involved in the experimental procedures prepared and labeled the syringes containing either ropivacaine or placebo. Syringes were identical in appearance and labeled according to the pregenerated randomization list, which was securely stored by this individual. The list remained inaccessible to the investigators during all experimental and data collection phases and was only disclosed after completion of all experiments to allow group allocation for statistical analysis. The RPVC group received ropivacaine 0.2% (ropivacaine hydrochloride, 2 mg/ml infusion solution; Sintetica, Germany), and the placebo group received normal saline (NaCl 0.9%; Fresenius Kabi, Germany). Both substances were administered *via* a regional anesthesia catheter (for detailed description, see “Anesthesia and Monitoring” and supplemental fig. 1 [https://links.lww.com/ALN/E406]). At the beginning of the experiment, 5 ml of the assigned study medication was administered perineurally, followed by a continuous infusion of 3 ml/h throughout the experiment (fig. 1).

### Anesthesia and Monitoring

On the day of surgery, the animals were fasted for 6 h before the induction of anesthesia. Premedication consisted of a mixture of tiletamine and zolazepam (Zoletil; Virbac, United Kingdom), and xylazine (Rompun 2%; Bayer, Austria), and butorphanol (0.3 mg/kg; VetViva Richter, Austria), administered subcutaneously. After intubation, anesthesia was maintained using inhalational sevoflurane (2 to 3%; Sevorane; AbbVie, Austria) alongside continuous infusions of fentanyl (0.018 mg · kg^−1^ · h^−1^; fentanyl-Hameln 50 µg/ml; Hameln Pharma, Germany) and rocuronium bromide (5 mg · kg^−1^ · h^−1^; rocuronium bromide Kabi 10 mg/ml; Fresenius Kabi).

Surgical access to the internal jugular vein and the common carotid artery was established, and intravascular catheters were placed in both vessels. A urinary catheter was also inserted. Vital parameters, including mean arterial pressure, heart rate, and pulse oximetry, were continuously monitored using an anesthesia monitoring system (Draeger Infinity Delta; Draegerwerk AG & Co., Germany). Blood gases, electrolytes, pH, base excess, glucose, and hemoglobin concentration were continuously monitored from arterial blood on a calibrated blood gas analyzer (ABL837 FLEX; Radiometer Medical, Denmark).

**Fig. 2.**
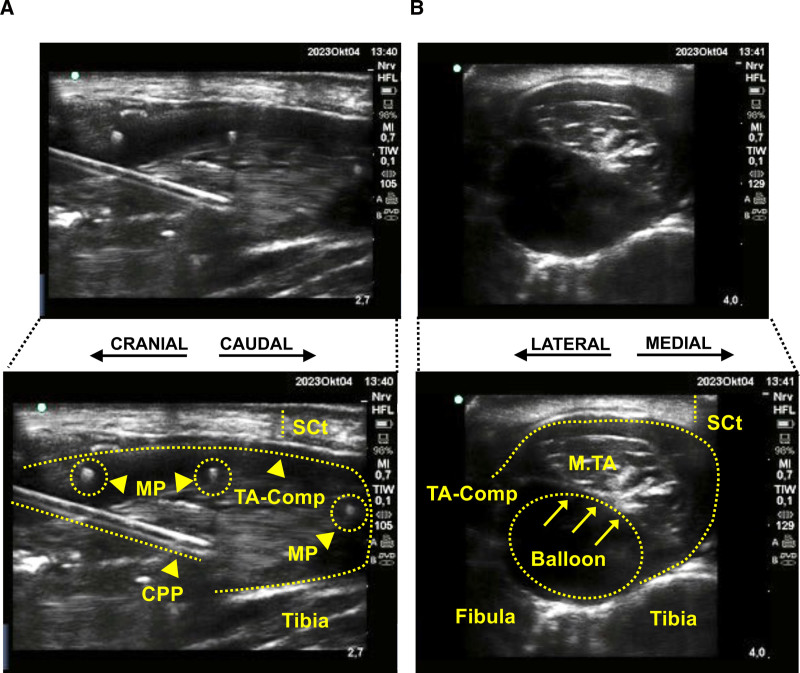
Ultrasound imaging depicting real-time imaging of the sagittal view of the tibialis anterior compartment (*A*) and the transversal view of the tibialis anterior muscle compartment (*B*). Balloon, angioplasty catheter; CPP, compartment pressure probe; MP, microperfusion probes; M.TA, tibialis anterior muscle; SCt, subcutaneous tissue; TA-Comp, compartment of the tibialis anterior muscle.

For measurement of compartment pressure, an 18-gauge needle was inserted into the tibialis anterior muscle of the right hind leg under ultrasound guidance (fig. 2) and connected to a pressure transducer (Draeger Infinity Hemomed; Draegerwerk AG & Co.). Pressure readings were continuously recorded and documented at predefined time points throughout the experiment (fig. 1).

A regional anesthesia catheter (Pajunk SonoLong Sono NanoLine Tuohy 18-gauge × 100-mm catheter; Pajunk, Germany) was placed adjacent to the right sciatic nerve under ultrasound guidance using a 14-MHz transducer (M-Turbo; Sonosite Ltd., USA; supplemental fig. 1, https://links.lww.com/ALN/E406). The administration protocol and timing relative to ACS induction are described under “Experimental Setup.” Administration of study medication followed the protocol described under “Randomization and Treatment Groups.”

### Compartment Syndrome Model

#### Experimental Setup

We implemented a modified version of the balloon-catheter model described by Kalns *et al.*,^24^ with refinements including elevated target pressures during the initiation phase and the use of a microperfusion probe for continuous interstitial sampling.^25^ Surgical access to the space between the interosseous membrane and the tibialis anterior compartment of the right lower leg was established, ensuring strict avoidance of fascia injury. A valvuloplasty catheter (VACS III PTV ballon-dilatational catheter, 26-mm diameter, 40-mm length; OSYPKA AG, Germany) was inserted into this space, and the catheter was secured using tight sutures before inflating the balloon (see supplemental video, https://links.lww.com/ALN/E407). The assigned study medication was administered after regional anesthesia catheter placement, followed in rapid succession by balloon inflation to induce ACS. The sequence and timing of catheter placement, block initiation, and ACS induction was identical in all animals to ensure comparability between groups. Compartment pressure was maintained at least 30 mmHg above mean arterial pressure for a duration of 6 h. The inability to maintain compartment pressure above mean arterial pressure (MAP; *e.g.*, due to balloon rupture or displacement) was defined as an exclusion criterion. As a result, one animal in the RPVC group was excluded from analysis after completion of the experiment due to balloon rupture, which prevented sustained achievement of the target compartment pressure. The setup and time course of the experiment are illustrated in figure 1. To maximize standardization, real-time ultrasound was used for the precise placement of both the angioplasty catheter and the intracompartmental pressure probe as additional modification to the setup of Kalns *et al.*^24^ Compartment pressure was measured using an optical pressure transducer connected to a digital recording system. The device was calibrated to external atmospheric pressure and inserted into the center of the tibialis anterior muscle using a 20-gauge intravenous catheter, placed at an approximately 25° angle to the skin surface. Representative ultrasound images of the anatomical placement of catheters and probes are shown in figure 2. Intramuscular (compartment) pressure was continuously monitored throughout the experiment. At the end of the observation period, tissue specimens of the tibialis anterior muscle were collected and processed for histopathologic evaluation (see “Histology”).

#### Study Outcomes

The primary outcome was the comparison of compartment pressure and the difference of MAP and compartment pressure (δ pressure) between specimens with and without local anesthetics administered through regional catheters. The primary outcome was assessed at T10 (2 h after balloon deflation). Secondary outcomes included levels of lactate, creatine kinase, glucose, and lactate dehydrogenase (LDH) in both plasma and interstitial fluid. Additionally, histologic findings were analyzed and scored.

### Analytical Methods

#### Plasma Analysis

At predefined time points (fig. 1), venous blood samples were collected into 3.5-ml tubes containing either buffered 3.2% trisodium citrate, ethylendiamintetraacetate, or lithium heparin (Vacuette; Greiner Bio-One, Austria) through the resting jugular vein catheter. Since all sampling was performed after surgical preparation and catheterization under deep anesthesia; no trauma-free pre-ACS baseline was feasible. The first measurement point (T1) reflects this combined procedural state. The lines were rinsed with saline, and a small blood volume was predrawn before sampling. The samples were immediately centrifuged at 2,500*g* for 15 min to obtain platelet-free plasma. A minimum of 550 µl of plasma was then carefully aliquoted into micronic cryotubes and stored at −80°C for further analysis. Creatine kinase, LDH, lactate, and glucose were determined on a clinical chemistry analyzer (Cobas C111; Roche AG, Switzerland). All other parameters were obtained from the continuous blood gas monitoring as described earlier.

#### Interstitial Fluid Analysis

To assess homeostatic changes in the affected muscle in comparison with the systemic circulation, interstitial fluid (creatine kinase) from within the tibialis anterior muscle compartment was collected *via* open flow microperfusion (OFM) and was sampled at corresponding blood withdrawal time points. OFM utilizes a membrane-free probe design that allows continuous sampling of interstitial fluid from various tissues.^26–30^

In our study, three OFM probes (OFM-PL3-75-LB dOFM three-channel pull tubing, MPP 102 PC; Joanneum Research, Austria) were used to collect interstitial fluid from the affected muscle compartment, with sampling points corresponding to those used for plasma analysis with a 20-min delay to complete collection. The probes were inserted under ultrasound guidance to ensure precise placement and depth within the muscle compartment (fig. 2). After rinsing the probes with transfer medium (Ringer’s lactate; Fresenius Kabi), continuous perfusion was maintained at a flow rate of 1 μl/min. Sampling volumes ranged between 120 and 180 μl/h, and fluids were frozen at −80°C immediately upon collection. Due to the complexity of the setup, OFM could only be performed in one animal at a time, and its allocation was randomized in the same manner as group assignment. To correlate the levels of interstitial markers and metabolic changes with the systemic circulation, creatine kinase, LDH, lactate, and glucose were determined on the same platform as the plasma samples (Cobas C111; Roche).

#### Histology

Upon the conclusion of the experiments, the animals were humanely euthanized. Immediately after, tissue samples were harvested to confirm presence of ACS by one skilled investigator (G.F.). Striated muscle tissue was carefully extracted from each subject and put in formaldehyde immediately. For comparative analysis, muscle tissue from the contralateral limb (without any intervention) was also collected in 5 of the 20 animals. A roughly 1-cm^3^ tissue block was extracted subfascially from the middle of the cranio-caudal line of the tibialis anterior compartment using a sterile scalpel. The samples were fixed in 10% buffered formalin, embedded in paraffin wax, and cut at a thickness of 2 µm. The sections were stained with hematoxylin and eosin and evaluated with a light microscope (Olympus BX53; Olympus Corp, Japan) by a veterinary pathologist blinded to treatment. For the semiquantitative evaluation, the scoring system by Kalns *et al.*^24^ was adapted, because the surviving time after insult was shorter in our study, and regeneration was not assessed. On a five-point scale (0 = not present, 1 = focal mild, 2 = multifocal mild, 3 = multifocal moderate, 4 = multifocal severe), the presence of the following lesions was assessed: muscle degeneration, muscle necrosis, edema, hemorrhage, and neutrophilic infiltration.

### Statistical Analysis

All of the data were compiled and organized using the Microsoft Office suite (Microsoft, USA). The sample size of n = 10 animals per group was established in accordance with ethical regulations and in agreement with the institutional and municipal animal welfare authorities. For comparisons between the dichotomous groups “RPVC” and “placebo,” Wilcoxon–Mann–Whitney tests were utilized to assess differences between the RPVC group and the placebo group across all measured variables. Effect sizes for these analyses were calculated using Cliff’s δ to provide a measure of the magnitude of observed effects. For histopathologic analyses, an additional reference was introduced by including measurements from the contralateral leg (*i.e.*, RPVC, placebo, and control). In this case, in which the independent variable “group” consisted of three categories, Kruskal–Wallis tests were employed, followed by *post hoc* Wilcoxon–Mann–Whitney tests with Bonferroni correction to account for multiple comparisons. All statistical analyses were performed using R version 4.1.2. Graphs and data visualizations were generated with GraphPad Prism version 5 (GraphPad Software, USA). The level of *P* < 0.05 was considered significant. The results are presented as median and interquartile ranges to account for nonnormal data distribution.

## Results

### Modified ACS Model

Based on guideline criteria, ACS was successfully induced in all animals.^31,32^ One subject in the RPVC group met exclusion criteria and was withdrawn from analysis. All assessed endpoint parameters are presented in tables 1 and 2.

**Table 1.** Pressure Readings and Analyte Concentrations at Each Time Point

Time Point	Treatment	Pressure Readings (Median [IQR]), mmHG			Concentrations (Median [IQR])							
		CP	δ Pressure	MAP	p-CK, U/l	p-Lac, mmol/l	p-LDH, U/l	p-Gluc, mg/dl	c-CK, U/l	c-Lac, mmol/l	c-LDH, U/l	c-Gluc, mg/dl
T1	Placebo	104 [90.2, 115]	−40.5 [−63.2, −30.0]	72.0 [68.5, 75.0]	1,273 [893, 1,378]	15.10 [12.27, 18.48]	568 [453, 588]	63.0 [52.2, 79.0]	238,140 [190,760, 295,840]	42.0 [41.0, 51.0]	11,450 [7,450, 13,250]	16.0 [13.0, 47.0]
	RPVC	115 [110, 118]	−40.0, [−50.0, −22.0]	68.0 [62.0, 80.0]	1,687 [1,167, 3,301]	14.80 [14.20, 23.30]	588 [560, 660]	71.0 [63.0, 86.0]	288,165 [190,290, 377,363]	60.5 [55.5, 67.8]	18,575 [11,900, 23,675]	32.5 [28.2, 33.8]
	*P* value	0.10	0.50	0.77	0.18	0.78	0.21	0.33	0.66	0.13	0.33	0.43
T3	Placebo	107 [91.2, 124]	−43.5 [−58.5, −29.5]	64.0 [60.0, 72.0]	1,131 [932, 1,262]	15.40 [14.10, 17.17]	501 [446, 564]	73.0 [64.0, 84.0]	56,780 [54,220, 57,260]	87.0 [77.0, 90.0]	3,450 [3,400, 4,100]	12.0 [5.0, 20.0]
	RPVC	115 [104, 125]	−46.0 [−49.0, −42.0]	66.0 [62.0, 72.0]	1,756 [1,028, 2,557]	13.70 [12.10, 18.20]	572 [549, 606]	74.0 [67.0, 93.0]	53,020 [28,310, 81,315]	100.0 [83.2, 156]	4,275 [2,700, 5,588]	3.0 [2.2, 3.8]
	*P* value	0.41	0.81	0.59	0.21	0.68	0.21	0.68	> 0.999	0.33	0.36	0.064
T6	Placebo	111 [94.0, 128]	−53.5 [−57.0, −45.2]	62.0 [59.2, 63.0]	950 [759, 1,186]	12.30 [12.03, 14.27]	469 [381, 511]	72.5 [55.5, 84.2]	155,220 [86,340, 244,280]	122 [113, 133]	11,650 [6,500, 18,500]	10.0 [7.0, 16.0]
	RPVC	115 [110, 119]	−42.0 [−49.0, −40.0]	64.0 [63.0, 69.0]	1,676 [878, 1,955]	12.80 [12.40, 14.10]	507 [470, 549]	78.0 [74.0, 87.0]	128,970 [75,645, 207,195]	178 [152, 233]	9,100 [5,950, 13,300]	7.5 [6.2, 11.8]
	*P* value	0.68	0.27	0.059	0.15	0.68	0.18	0.44	0.66	0.082	0.79	0.58
T8	Placebo	35.0 [29.2, 42.2]	22.0 [15.2, 31.8]	60.0 [56.8, 61.8]	1,053 [803, 1,211]	12.70 [11.40, 14.53]	467 [389, 517]	71.0 [67.0, 82.2]	1,024,400 [452,160, 1,065,200]	218 [175, 237]	71,950 [32,050, 115,400]	46.0 [40.0, 54.0]
	RPVC	47.0 [39.0, 51.0]	21.0 [16.0, 26.0]	63.0, [61.0, 70.0]	1,733 [803, 2,111]	13.50 [12.50, 14.30]	482 [467, 609]	79.0 [70.0, 88.0]	735,300 [585,800, 2,575,225]	243 [150, 310]	38,600 [27,225, 122,238]	24.0 [22.2, 42.2]
	*P* value	0.050	0.65	0.065	0.31	0.51	0.21	0.25	0.93	0.71	> 0.999	0.13
T10	Placebo	41.5 [38.2, 47.2]	11.5 [7.2, 17.2]	59.0 [56.2, 61.8]	1,171 [1,020, 1,467]	11.55 [11.07, 15.12]	456 [397, 497]	76.0 [49.0, 83.2]	495,600 [493,720, 908,600]	168 [160, 237]	113,800 [91,000, 116,600]	51.0 [43.0, 103]
	RPVC	57.0 [49.0, 59.0]	9.0 [6.0, 17.0]	63.0 [58.0, 65.0]	1,940 [868, 3,334]	12.50 [11.40, 14.50]	493 [476, 568]	73.0 [64.0, 83.0]	368,700 [244,020, 444,600]	156 [87.5, 194]	24,475 [19,500, 28,213]	19.5 [11.5, 27.5]
	*P* value	0.022	0.62	0.13	0.24	0.68	0.29	0.81	0.43	0.54	0.017	0.0043

The data are presented as medians with interquartile ranges (IQRs). “δ Pressure” indicates the change in pressure (MAP – CP).

c-, compartment interstitial fluid; CK, creatine kinase; CP, compartment pressure; Gluc, glucose; Lac, lactate; LDH, lactate dehydrogenase; MAP, mean arterial pressure; p-, plasma; RPVC, ropivacain.

**Table 2.** Histologic Findings at Time Point 10

Treatment	Histologic Findings											
	Degeneration	Necrosis	Regeneration	Edema/Fibrin	Hemorrhage	Mineralization	Microthrombi	Neutrophils	Eosinophils	Mononuclear	Mixed	Vasculitis
Placebo	3.0 [2.2, 4.0]	0.5 [0, 1]	0 [0, 0]	0 [0, 1]	1 [1, 2]	0 [0, 0]	0 [0, 0]	0.5 [0, 1.8]	0 [0, 0]	0 [0, 0]	1 [0, 1]	1 [0, 1]
RPVC	1 [1, 1]	0 [0, 0]	0 [0, 0]	0 [0, 0]	1 [0, 2]	0 [0, 0]	0 [0, 0]	0 [0, 1]	0 [0, 0]	0 [0, 0]	0 [0, 1]	0 [0, 0]
*P* value	0.0018	0.020	NA	0.22	0.39	NA	NA	0.36	NA	NA	0.26	0.032

The data are presented as medians with interquartile ranges.

NA, not applicable; RPVC, ropivacaine.

**Fig. 3.**
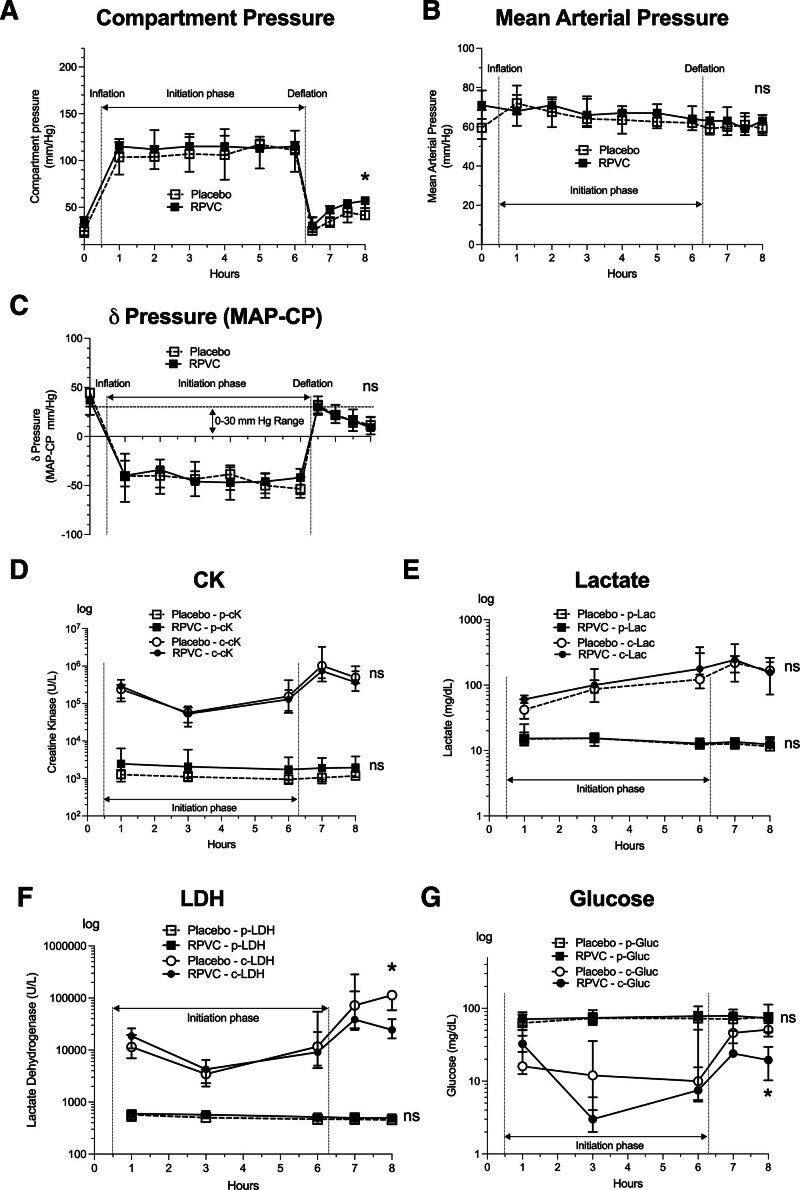
Pressure readouts and metabolic profile in plasma and within the compartment. (*A*) Compartment pressure (CP). (*B*) Mean arterial pressure (MAP). (*C*) δ Pressure (MAP − CP). The *dashed lines* indicate the 30-mmHg consensus range for ACS definition. Readings are scaled in millimeter mercury (mm Hg). The data are plotted as medians with interquartile ranges (IQR). The trajectories of tissue damage markers and selected metabolites in the plasma and intracompartmental interstitium of animals enrolled in the animal model of acute compartment syndrome (ACS). (*D* to *G*) Concentrations of creatine kinase (*D*, CK), lactate (*E*), lactate dehydrogenase (*F*, LDH), and glucose (*G*) are given on a logarithmic scale (log10) at the corresponding sampling points to indicate the magnitude of difference between plasma (p-) and intracompartmental (c-) levels of the respective parameters on the same axis. *, significant (*P* < 0.05); ns, nonsignificant; RPVC, ropivacaine.

After deflation of the angioplasty balloon, compartment pressure increased in both groups. At T8, compartment pressure reached a median of 35.0 mmHg (interquartile range [IQR], 29.2 to 42.2) in the placebo group and 47.0 mmHg (IQR, 39.0 to 51.0) in the RPVC group (*P* = 0.050). By T10 (2 h postdeflation), compartment pressure rose further to 41.5 mmHg (38.2 to 47.2) in placebo *versus* 57.0 mmHg (49.0 to 59.0) in RPVC (*P* = 0.022), reflecting a 15.5-mmHg difference between groups (fig. 3A). However, once normalized for systemic blood pressure (*i.e.*, δ pressure = compartment pressure − MAP), the difference was no longer significant (fig. 3C). MAP remained comparably stable over time in both groups, exemplified at T10 by median values of 59.0 mmHg (56.2 to 61.8) in placebo and 63.0 mmHg (58.0 to 65.0) in RPVC (*P* = 0.13; fig. 3B).

### Tissue-damage and Metabolic Markers in the Compartment and Plasma Measurements

At T1, all plasma and interstitial biomarkers showed no significant differences between RPVC and the placebo group, confirming group comparability at initiation. Induction of ACS was not associated with significant fluctuations in the plasma concentration of creatine kinase, a marker of skeletal muscle damage. Although the overall median plasma creatine kinase (p-CK) levels were higher in the RPVC group than in the placebo group—for example, at T1, p-CK was 1,687 U/l (IQR, 1,167 to 3,301) in RPVC *versus* 1,273 U/l (IQR, 893 to 1,378) in placebo, and at T10, p-CK was 1,940 U/l (IQR, 868 to 3,334) in RPVC compared to 1,171 U/l (IQR, 1,020 to 1,467) in placebo—these differences did not reach statistical significance (*P* > 0.05; fig. 3D).

Creatine kinase levels measured within the tissue compartment (c-CK) showed only slight fluctuations during the initiation phase and were markedly elevated relative to plasma levels throughout the experiment. For instance, at baseline (T1) in the placebo group, the median p-CK was 1,273 U/l, whereas the median c-CK was 238,140 U/l—representing an approximately 187-fold difference. Similarly, in the RPVC group at T1, median p-CK was 1,687 U/l compared to a c-CK of 288,165 U/l (roughly 171-fold higher). Nonetheless, neither the absolute nor the recirculation levels of c-CK differed significantly between RPVC and placebo (*P* > 0.05; fig. 3D).

Plasma lactate concentrations remained relatively invariant throughout the experiment, with median values ranging from approximately 11.55 to 15.40 mM and no significant intergroup differences. In contrast, the lactate concentration within the tissue compartment (c-Lac) increased over time in both groups; for example, in the RPVC group, c-Lac rose from 60.5 mM (IQR, 55.5 to 67.8) at T1 to 156 mM (IQR, 87.5 to 194) at T10 (fig. 3E).

Plasma lactate dehydrogenase levels were stable in both groups. Conversely, compartment LDH (c-LDH) followed a pattern analogous to that of creatine kinase but surged to significantly higher levels in the placebo group after deflation; at T10, c-LDH was 113,800 U/l (IQR, 91,000 to 116,600) in the placebo group compared to 24,475 U/l (IQR, 19,500 to 28,213) in RPVC (*P* = 0.017; fig. 3F).

All animals maintained a physiologic concentration of plasma glucose until the end of the experiment. In contrast, tissue compartment glucose (c-Gluc) decreased from its physiologic level and was markedly lower in the RPVC group compared to placebo during the initiation phase. The RPVC group reached a nadir at T3 with c-Gluc levels of 3.0 mg/dl (IQR, 2.2 to 3.8) *versus* 12.0 mg/dl (IQR, 5.0 to 20.0) in placebo and remained lower at T10 (RPVC: 19.5 mg/dl [IQR, 11.5 to 27.5] *vs*. placebo: 51.0 mg/dl [IQR, 43.0 to 103.0]; *P* = 0.0043) despite a transient increase between T6 and T8 (fig. 3G).

### Histologic Characterization of the Compartment Region

**Fig. 4.**
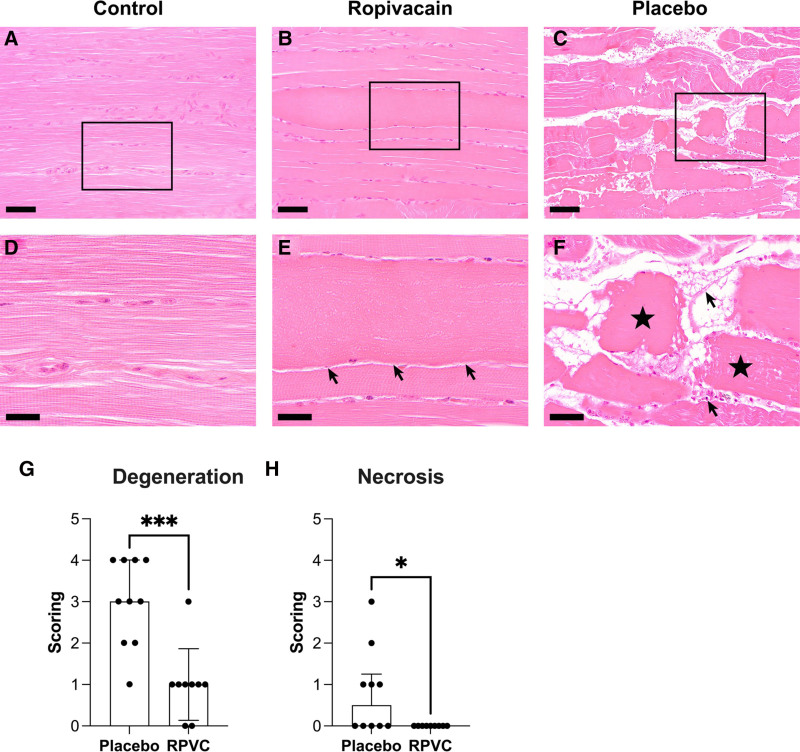
(*A* to *F*) Representative images of histologic lesions in longitudinal muscle sections. (*A*, *D*) Physiologic structure of muscle in control samples with regular striation of myofibers. (*B*, *E*) Degeneration of one myofiber with swollen hyalinized sarcoplasm and loss of striation in an animal of the ropivacain (RPVC) group (*arrows* denote the degenerate fiber). (*C*, *F*) Fragmentation (*stars*) of necrotic myofibers with extravasation of erythrocytes, single neutrophils, and fibrin (*arrows*) in an animal of the placebo group (hematoxylin and eosin staining). *Bars* indicate 50 µm (*A* to *C*) or 20 µm (*D* to *F*). (*G*, *H*) Comparison of histologic scores between groups for degeneration (*G*) and necrosis (*H*). *, significant (*P* < 0,05); ***, highly significant (*P* < 0.001; Mann–Whitney test).

All animals showed histologic lesions to some extent on the limb where ACS was initiated. Degenerating myofibers, characterized by swollen hyalinized sarcoplasm with loss of striation and pycnotic nuclei (fig. 4, B and E), were detectable in all but two animals of the RPVC group and all animals of the placebo group. Necrosis with fragmentation of myofibers and nuclei (fig. 4, D and F) was detected in six animals of the placebo group, but not in the RPVC group. Edema was present in all animals after ACS in a mild to moderate extent. Scores for degeneration, necrosis, and edema were significantly increased in the placebo group compared to control, but not in the RPVC group compared to control. Furthermore, scores for necrosis were significantly increased in the placebo group compared to the RPVC group (table 2). Extravascular erythrocytes and neutrophils were present predominantly in the interstitial tissue of most of the animals after ACS initiation. Lesions were focally or multifocally distributed. Figure 4 shows representative images of all groups.

## Discussion

In this porcine model of ACS, continuous regional anesthesia with ropivacaine was associated with elevated compartment pressure but a more favorable metabolic profile and reduced markers of muscle injury. These findings suggest a potential protective effect of perineural ropivacaine against ischemia-induced tissue damage and support further investigation of regional anesthesia in ACS despite the presence of an elevated compartment pressure. In our study, we reproduced and further developed the preclinical ACS model originally described by Kalns *et al*.^24,25^ Dynamics of compartment pressure and δ pressure in the current setup also showed similarity to earlier models of ACS.^9,33,34^ Our most meaningful technical expansion was the use of a microperfusion probe in the affected compartment facilitating a repeated collection of creatine kinase. This enabled us to monitor the local release of cell- and tissue-damage markers and metabolites and compare site-specific release profiles with their concentrations in the systemic circulation. Thus, the use of the probe provided a deeper insight into the regional anesthesia–mediated effects in the ACS micromilieu and related compartment pressure readings with the actual damage to skeletal muscle. To our knowledge, the current study was the first to use OFM in the muscle tissue. In contrast to creatine kinase, for which no reference values are reported for the assessed parameters, the direction and magnitude of plasma biomarker trends observed in our model align with previously established ranges in large animal studies and the clinical literature.^35^ In contrast to Kalns *et al*.,^24^ we applied a higher target compartment pressure during the initiation phase (compartment pressure greater than or equal to 30 mmHg above MAP) that translated into a higher compartment pressure in the edema phase compared to earlier ACS pig studies.^25,36–39^

Given that pain represents the earliest and most common symptom of ACS, regional anesthesia would not only interfere with clinical diagnostics but also affect pressure-based criteria.^11,40^ Intracompartmental pressure (ICP) monitoring has shown high sensitivity (94%) and specificity (98%) when using a differential pressure threshold of 30 mmHg for more than 2 h.^41,42^ Thus, when clinical suspicion is high, ICP monitoring is considered the gold standard in supporting diagnosis. While various other diagnostic techniques have been studied (*e.g.*, oxygenation, perfusion, biomarker measurements), none have yet surpassed pressure monitoring in effectiveness.^8,36,38,39,43,44^ Notably, a recent large clinical study found no evidence that regional anesthesia increases the risk of missed ACS, reinforcing the feasibility of regional anesthesia use with appropriate monitoring. Interestingly, the authors also observed lower rates of ACS diagnosed during hospitalization in the regional anesthesia group, which they attributed to potential confounding rather than a causal relationship.^45^ However, referring to compartment pressure as an independent ACS marker during regional anesthesia may overlook the potential benefits of an increased perfusion pressure in the preservation of vascular patency to the affected tissue. For instance, it was shown in a small series of controlled volunteer studies that the perfusion index (ratio of pulsatile to nonpulsatile blood flow) shows a 2-fold increase after peripheral nerve blocks.^20–22^

By reversibly inhibiting sodium ion influx in nerve fibers, RPVC not only produces effective sensory blockade with less motor blockade than bupivacaine, but it also exhibits complex vascular effects.^46,47^ RPVC also induced a dose-dependent vasoconstriction in the mouse mesenteric arteries, partially mediated by the endothelium and cyclooxygenase.^48^ In the guinea pig aorta, RPVC showed a biphasic effect: vasoconstriction at low concentrations and vasorelaxation at high concentrations.^49^ When used for digital nerve blocks, RPVC with epinephrine typically maintains an adequate skin perfusion and provides prolonged pain reduction compared to other local anesthetic formulations.^50^

Both markers of cellular decay, c-LDH and the muscle-specific c-CK, showed U-shaped trajectories throughout the initiation phase. Kalns *et al*.^24^ attributed this to the initial surgical preparation, clearance effects and a subsequent, continuous increase toward deflation of the balloon. Compartment pressure steadily rose after balloon deflation, indicating a progressing degeneration and edema formation. During this passive phase of reperfusion, c-CK and c-LDH showed similar dynamics in both groups, with LDH reaching significantly lower levels 2 h after deflation. Considering a suspected, higher perfusion index in regions treated with RPVC, reperfusion, oxygen supply, and the clearance of metabolites associated with ischemia and degradation might also occur at a higher pace. Lower c-LDH at the end of observation therefore also suggests that the hemodynamic effects of RPVC mitigated ischemia–reperfusion injury as a consequence of improved circulation within the compartment. Interestingly, c-Lac, a byproduct of anaerobic metabolism steadily increased in both groups and was only marginally affected by the deflation of the balloon. At the same time, glucose consumption was higher in the RPVC group during initiation of ACS and again reached significantly lower levels 2 h after deflation. Doro *et al.*^43^ showed a decrease of glucose in a canine ACS model, concluding that its decrease represented a robust marker of an incipient ACS. The similar c-Lac increase with the concurrently lower glucose in the RPVC group we observed likely indicates a shift from glycolysis to oxidative phosphorylation.

The simultaneous decrease in glucose and the stabilization or increase in lactate points to an efficient glucose metabolism pathway that favors energy production and tissue regeneration. Lower c-Gluc levels in the RPVC group may indicate that the glucose is being diverted from glycolysis and lactate production toward oxidative phosphorylation and regenerative biosynthetic pathways, including adenosine triphosphate generation and cellular repair mechanisms. This is consistent with findings that lactate oxidation supports recovery and regeneration in energy-deprived or ischemic tissues, suggesting that tissues with better oxygen and glucose perfusion may be more adept at utilizing these substrates for energy and repair.^51,52^ However, these observations on a metabolic level required confirmation through histologic assessment of the muscle tissue around the same time point.

At the end of the experiment, 2 h after deflation, histologic examinations showed a greater extent of tissue damage in the placebo group. This effect was significant in some of the examined categories such as degeneration and necrosis but was inconclusive in other histologic outcome parameters. These findings substantiate our hypothesis that regional anesthesia raises both systolic and diastolic blood flow and that circulation is increased in dependent anatomic areas. In consequence, oxygen supply is likely also enhanced by increased perfusion of the affected limbs after regional anesthesia.

### Limitations

We acknowledge several limitations in the study. First, we tested a single anesthetic agent at a single concentration (RPVC 2 mg/ml), limiting the generalizability of our findings. Second, the short duration of the experiment limits conclusions to the acute ACS sequelae and a potential early therapeutic benefit with a late deterioration that would require follow-up time points after successfully recovering the animals. Although occurring in rapid succession, the administration of study medication and the induction of ACS did not follow the typical sequence in clinical practice, in which regional anesthesia is usually performed after the injury has occurred. Furthermore, because regional anesthesia and ACS induction were initiated immediately after catheter placement, we were unable to obtain a completely trauma-free baseline. All sampling therefore began after surgical preparation and catheterization under deep anesthesia. While published reference ranges for porcine plasma biomarkers (*e.g.*, lactate, glucose, LDH, creatine kinase) suggest that our T1 values were modestly elevated as expected after anesthesia and instrumentation, no significant intergroup differences were detected at T1.^35^ This supports comparability, but the absence of a trauma-free baseline remains a limitation when interpreting early biomarker dynamics. Furthermore, while compartment pressure is often compared to diastolic pressure in clinical settings, our study followed the model described by Kalns *et al.*,^24^ using MAP as a reference. Due to technical constraints, diastolic pressure was not continuously recorded, preventing direct comparison to clinical δ pressure thresholds. As only male pigs were used to model the clinical population most affected by ACS, our findings may not be generalizable to females, in whom hormonal differences could influence ischemia–reperfusion injury or drug effects.

Given the small sample size and nonnormal distribution of several variables, we analyzed repeated measurements using nonparametric pairwise comparisons rather than mixed-effects models; this approach may have reduced statistical power and did not account for within-subject correlation. Finally, the exact mechanisms underlying the observed metabolic shifts/muscle protection remain speculative and warrant further investigation.

### Conclusions

Using a modified porcine model of ACS, this study provides novel insights into the effects of regional anesthesia on ACS progression. Although ICP readings were higher in the regional anesthesia group, normalization to systemic pressures eliminated differences, highlighting improved perfusion rather than exacerbated ischemia. While plasma markers such as creatine kinase, LDH, and glucose remained stable, interstitial fluid analysis revealed dynamic changes. Regional anesthesia–treated animals exhibited reduced glucose levels, suggesting enhanced oxidative metabolism, and lower LDH levels during reperfusion, indicating mitigated ischemia–reperfusion injury. Histologic findings further supported this, showing reduced necrosis and degeneration in regional anesthesia–treated animals. These results challenge traditional concerns about regional anesthesia in ACS by demonstrating its potential to enhance tissue perfusion and mitigate ischemic damage without exacerbating injury. Our observations not only indicate that regional anesthesia does not have a detrimental effect on cellular decay during ACS, but they also demonstrate the accuracy of compartment pressure measurement with regard to degeneration and necrosis of muscle tissue. While additional studies are needed to explore long-term outcomes and diagnostic implications, this work establishes regional anesthesia as a potential modulator of ischemic injury in ACS and a foundation for more nuanced clinical applications.

### Acknowledgments

The authors express profound gratitude for the dedication and tireless efforts of technical personnel and support staff involved in conducting this study. They especially thank the experimental surgery group at LBI Trauma (Vienna, Austria) for support in preparing and performing the experiments. The authors are grateful to Joanneum Research (Graz, Austria) for support in the application of microperfusion. The authors sincerely appreciate Philipp Lirk, M.D. (Brigham and Women’s Hospital, Harvard Medical School, Boston, Massachusetts), for the inspiration to conduct this study. Finally, the authors thank Elisa Kuen, B.Sc, Karl Kropik, Anastasia Moschou, M.D, and Carina Wagner at LBI Trauma for invaluable technical support.

### Research Support

This study was funded by the Austrian Science Fund, Vienna, Austria (FWF, project number P-35425). The researchers received financial support from the Austrian Worker’s Compensation Board (AUVA), Vienna, Austria. The article processing charge was covered by institutional funds provided by the Ludwig Boltzmann Institute for Traumatology, Vienna, Austria. For the purpose of open access, the authors have applied for a CC BY public copyright license to any author-accepted article version arising from this submission.

### Competing Interests

The authors declare no competing interests.

### Supplemental Digital Content

Supplemental Figure 1. Ultrasound-guided ischiadic block placement, https://links.lww.com/ALN/E406

Supplemental Video 1. Insertion of angioplasty catheter in lower extremities, https://links.lww.com/ALN/E407

## Supplementary Material
