# Supplementary material for: Ropivacaine-based Regional Anesthesia Exerts Muscle-protective Effects Despite Elevated Compartment Pressure in a Porcine Model of Acute Compartment Syndrome
Source: Anesthesiology. 2026 Jan 23;144(5):1199–213. doi: 10.1097/ALN.0000000000005950 (PMC13064834; doi:10.1097/ALN.0000000000005950)
Supplement: Supplementary file 2 [file aln-144-1199-s002.docx]

Supplementary Video 1: Insertion of angioplasty catheter in lower extremit
